# A Core–Shell Elastic Flame Retardant with Superior Migration Resistance for Fire-Safe and Toughened Polyamide 66

**DOI:** 10.3390/polym18030363

**Published:** 2026-01-29

**Authors:** Jingfan Zhang, Xiao-Jie Li, Guowen Ran, Xiaoting Fu, Haisheng Xie, Xiangtian Yu, Chaofeng Chen

**Affiliations:** 1 China Bluestar Chengrand Co., Ltd., Chengdu 610041, China; zhangjingfan@sinochem.com (J.Z.); lixiaojie7@sinochem.com (X.-J.L.); ranguowen@sinochem.com (G.R.); fuxiaoting@sinochem.com (X.F.); xiehaisheng@sinochem.com (H.X.); 2 National Engineering Research Centre for Structure Plastic, Chengdu 610041, China

**Keywords:** Polyamide 66, flame retardancy, migration resistance, polymethylvinylsiloxane

## Abstract

A major challenge for halogen-free flame retardants is their tendency to migrate under high-temperature and high-humidity environments. For instance, the combination of aluminum diethylphosphinate (ADP) and melamine polyphosphate (MPP) used in polyamide 66 (PA66) easily migrated to the surface, leading to a white and frost-like appearance. To address this issue, a core–shell elastic flame retardant (SiR@FR) was prepared via a solution deposition method, wherein a polymethylsiloxane (SiR) layer was encapsulated on the surface of ADP and MPP. This shell not only improved the hydrophobicity of the FR but also the toughness of PA66. Experimental results demonstrated that PA66 with 9-SiR@FR (PA66-5) exhibited excellent migration resistance, with no visible surface whitening after 480 h of aging at 85 °C and 85% relative humidity. Meanwhile, PA66-5 displayed outstanding flame retardancy, achieving a UL-94 V-0 rating with an approximate 65% decrease in peak heat release rate compared with control PA66. Furthermore, SiR@FR enhanced the toughness of PA66 by alleviating stress concentration, resulting in a 21% increase in impact strength. This study presents a simple but reliable encapsulation strategy for fabricating flame-retardant PA66 composites that combine superior migration resistance and satisfactory mechanical properties, showing promising potential for demanding applications requiring long-term usability and stability.

## 1. Introduction

Polyamide 66 (PA66) is a high-performance engineering thermoplastic valued for its outstanding mechanical properties, thermal stability, and chemical resistance, which have led to its widespread adoption in the automotive, electrical, and aerospace industries [1,2,3,4]. Despite these advantages, its inherent flammability presents a significant safety hazard, restricting its applications where stringent flame retardancy is required [5,6].

Phosphorus-based flame retardants (FRs), including red phosphorus [7,8,9], melamine cyanurate (MCA) [10,11], aluminum diethylphosphinate (ADP) [12,13], DOPO [14,15,16], and melamine pyrophosphate (MPP) [17,18,19], have attracted considerable interest as promising candidates for halogen-free FRs owing to their more favorable environmental profile and effective flame-retardant performance [20,21]. In particular, the integration of ADP and MPP is recognized as one of the most efficient halogen-free FRs for polyamides. It offers an optimal balance between high flame-retardant efficiency and minimal degradation of mechanical properties, especially for glass-fiber-reinforced PA66 composites [22,23,24,25]. 

Nevertheless, this improved flame retardancy is frequently compromised by a persistent and practical issue: the flame retardants tend to migrate to the polymer surface under high-temperature and high-humidity conditions (e.g., 85 °C and 85% relative humidity). This blooming phenomenon is often visually identified as whitening or frosting on the surface of the polymers [26,27,28,29]. It leads to a progressive loss of flame retardancy, and the blooming of the FRs results in accelerated corrosion of the adjacent metal electronic components. This process substantially reduces the operational lifespan of flame-retardant electronic components [30].

Regarding the issue of FRs blooming, several strategies such as the use of reactive FRs, encapsulation techniques, and compatibilizers have been explored to mitigate blooming by enhancing interfacial adhesion and creating a physical barrier that hinders diffusion to the surface [31,32,33,34,35,36,37,38]. However, to the best of our knowledge, the available literature focuses on general-purpose plastics with low processing temperature, such as polypropylene and ethylene vinyl acetate copolymer [39,40,41]. The issue of blooming persists in engineering plastics with high processing temperatures, especially for hydrophilic PA66. The demands for improved thermal and shear stability have translated into more stringent application requirements for modified FRs used in PA66 [42,43,44].

This study proposes a strategy that employs thermally stable, hydrophobic, and elastic polymethylsiloxane (SiR) as the shell for the FRs (the mass ratio of ADP and MPP was 3:1) to mitigate their migration. Vinyl groups worked as cross-linking sites, improving the thermal stability and shear stability of the SiR. The Si-O-Si linkage accounted for the excellent hydrophobicity and elasticity, which provided the FRs with migration resistance and flame-retardant PA66 with improved toughness, respectively. The influence of SiR on flame-retardancy, mechanical performance, and migration resistance was systematically investigated. Furthermore, the mechanism of the flame retardancy and FRs migration resistance was elucidated. This work provides both theoretical insight and practical value for the development of high-performance and flame-retardant PA66 composites.

## 2. Experimental Section

### 2.1. Materials

Polyamide 66 (PA66, EP1107, MFR177) was purchased from Huafon Chemical Co., Ltd. (Wenzhou, China). Methylvinylpolysiloxane (MVPS), 2,5-Dimethyl-2,5-di(tert-butylperoxy)hexane (DHBP-95) and black master batch were provided by China Bluestar Chengrand Co., Ltd. (Chengdu, China). Aluminum diethylphosphinate (ADP), melamine polyphosphate (MPP), and ethyl acetate were procured from Shanghai Macklin Biochemical Co., Ltd. (Shanghai, China). Glass fibers (GF, ECS3014B) were purchased from Chongqing Polymercomp Co., Ltd. (Chongqing, China). 1,6-Bis-(3,5-di-tert-butyl-4-hydroxyhydrocinnamido)-hexane (Antioxidant 1098) and Tris(2,4-di-tert-butylphenyloxy)phosphine (Antioxidant 168) were provided by BASF SE (Ludwigshafen, Germany).

### 2.2. Preparation of SiR@FR

**Scheme 1:**
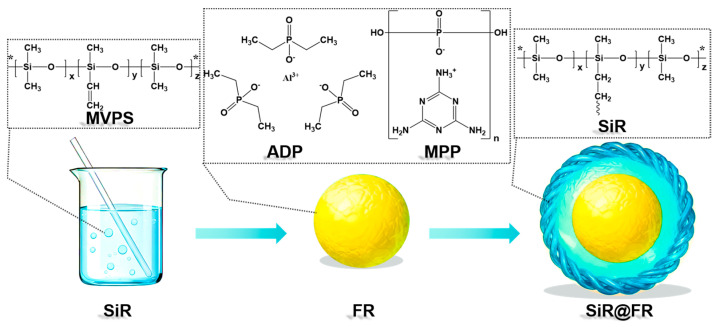
Synthesis route of x-SiR@FR.

A certain amount of MVPS and DHBP-95 as initiators was sequentially added to a beaker containing ethyl acetate (100 mL). The mixture was stirred at ambient temperature until completely dissolved and homogenized. Subsequently, this homogeneous mixture was blended with flame retardants consisting of aluminum diethylphosphinate (ADP, 150 g) and melamine polyphosphate (MPP, 50 g). The mixture was stirred at 1000 rpm with a high-speed mixer continuously at ambient temperature for 5 min to ensure uniform dispersion. Finally, this mixture was placed in a vacuum oven at 120 °C for 2 h to remove the solvent and complete the curing reaction. The resulting product was then milled to obtain the polysiloxane-encapsulated FRs (x-SiR@FR, where x represents the mass fraction of SiR in the mixture of ADP and MPP, with values of 3, 6, and 9, respectively). The corresponding synthetic pathway and formulas for x-SiR@FR are depicted in Scheme 1 and Table 1.

### 2.3. Preparation of Flame-Retardant PA66 Composites

Prior to processing, PA66 pellets were dried in an oven at 120 °C for 24 h. Subsequently, according to the formulations listed in Table 2, the dried PA66 and x-SiR@FR were pre-mixed with other raw materials. After thorough mixing, the premix was fed into a twin-screw extruder (CTE 35, Coperion, Nanjing Machinery Co., Ltd., Nanjing, China) with a screw speed of 250 rpm, a screw L/D ratio of 42:1, and melt compounding with the extrusion temperature at 250, 270, 270, 270, 260, 250, 250, 250, 250, and 270 °C, respectively. During extrusion, glass fibers were precisely metered into the system via a side-feeder gravimetric dosing device at the middle of the screw. Finally, standard test specimens were injection-molded from the dried pellets at a melt temperature ranging from 250 to 260 °C with an injecting molding machine (HTF90W1, Haitian Plastics Machinery Group Co., Ltd., Ningbo, China). The obtained samples were denoted as PA66-1, PA66-2, PA66-3, PA66-4, and PA66-5. Among them, PA66-1 and PA66-2 were samples without the addition of x-SiR@FR.

### 2.4. Characterization

Fourier-transform infrared (FTIR) spectra were acquired on a Nicolet IS50 spectrometer (Thermo Scientific, Waltham, MA, USA) across the spectral region of 500–4000 cm^−1^.

Surface morphological features of the FR and SiR@FR were observed with a Regulus 8100 scanning electron microscope (SEM, Hitachi, Tokyo, Japan), and an energy-dispersive X-ray spectrometer (EDS) was used to measure the distribution of elements. Before imaging, all samples were gold-sputtered.

The particle size of the sample was measured using a laser particle size analyzer (RISE-2002, Jinan Runzhi Technology Co., Ltd., Jinan, China) after dispersing 1.0 g of the material in 100 mL of water under ultrasonication for 1 min, following a standardized and repeatable testing procedure.

X-ray photoelectron spectroscopy (XPS) was conducted with a ESCALab 250Xi instrument (Thermo Scientific, Waltham, MA, USA) under high vacuum (4 × 10^−9^ mbar), using an Al Kα X-ray source.

Thermogravimetric analysis (TGA) was carried out on a TGA5500 instrument (TA Instruments, New Castle, DE, USA) with sample masses of 5 mg. The temperature was ramped from 40 to 800 °C at 10 °C/min under nitrogen atmosphere.

Flame retardancy properties were evaluated through the limiting oxygen index (LOI) test and vertical flame test (VFT) according to ASTM D2863 [45] and ASTM D3801 [46], respectively, using TESTech apparatus (Testech Group Co., Ltd., Suzhou, China). Sample dimensions were 130 × 6.5 × 3.2 mm^3^ for the LOI and 130 × 13 × 1.6 mm^3^ for the VFT. The LOI test and VFT were repeated 5 times for each sample. Cone calorimetry (CCT) was performed following ISO 5660 [47] under 50 kW m^−2^ radiant flux on 100 × 100 × 3 mm^3^ specimens, and it was repeated 3 times for each sample.

The contact angle was measured at ambient temperature using an optical contact angle goniometer (Dataphysics OCA20, Stuttgart, Germany). A deionized water droplet of approximately 3 μL was dispensed onto the sample surface, and the static contact angle was determined by analyzing the droplet profile. For each sample, measurements were performed at five different locations, and the average value is reported.

Tensile tests were conducted on an Instron 5967 testing system (Instron, Norwood, MA, USA) according to ISO 527-1:2012 [48]. Flexural properties were determined via three-point bending tests on a SUNS UTM5305 universal testing machine (Shenzhen, China), following ISO 178:2019 [49]. Notched Izod impact strength was measured with an EPY7400E pendulum impact tester (Shenzhen, China) in compliance with ASTM D256 [50]. The mechanical tests were repeated 5 times for each sample.

## 3. Results and Discussion

### 3.1. Characterization of SiR@FR

**Figure 1:**
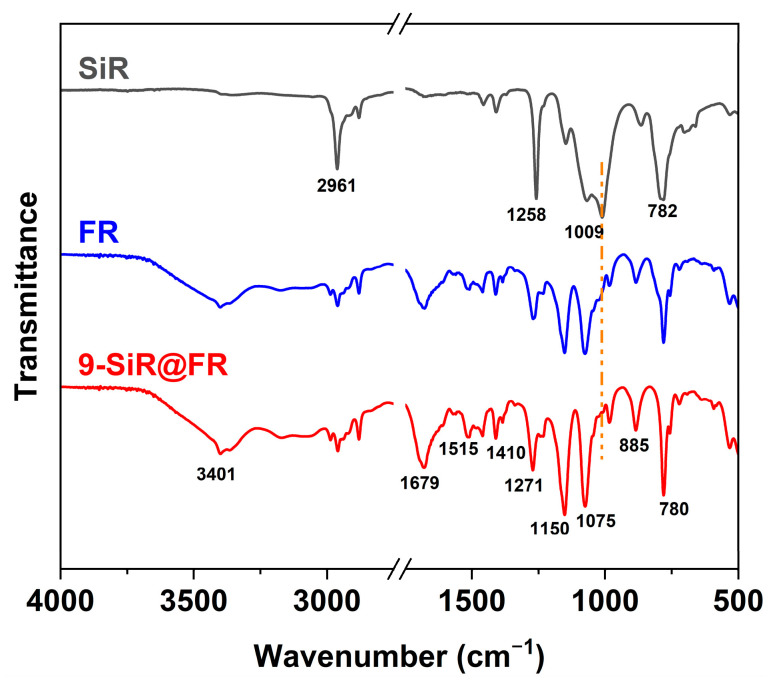
The FTIR of the SiR, FR, and 9-SiR@FR.

Figure 1 presents the FTIR spectra of SiR, FR, and 9-SiR@FR. In the spectrum of SiR, the absorption peak at 1009 cm^−1^ was ascribed to the asymmetric stretching vibration of Si−O−Si. The peak at 782 cm^−1^ corresponded to the Si−C stretching vibration, while the sharp peak at 1258 cm^−1^ arose from the symmetric deformation vibration of Si−CH_3_ [51,52]. The spectrum of 9-SiR@FR retained the characteristic peaks corresponding to Si−O−Si at 1009 cm^−1^ of SiR and distinctly exhibited absorption peaks attributable to the FR. Peaks at 3401 cm^−1^ and 1679 cm^−1^ originated from N−H stretching vibrations. The peak at 1515 cm^−1^ was assigned to C=N stretching, and the peak at 1410 cm^−1^ stemmed from C−N stretching vibrations. Notably, peaks at 1271 cm^−1^ and 1150 cm^−1^ corresponded to P=O and P−O stretching vibrations, respectively, and the absorption peak at 1075 cm^−1^ was attributed to P−OH stretching vibrations. Furthermore, the peak at 885 cm^−1^ corresponded to P−O−P symmetric and asymmetric stretching vibrations, and the feature at 780 cm^−1^ was P−C stretching vibrations. No new characteristic absorption peaks were observed in all of the aforementioned spectra, indicating the absence of strong chemical interactions between the SiR and FR. Furthermore, the Si−O−Si peak at 1009 cm^−1^ showed no significant shift compared to that of pure SiR. Therefore, the interfacial interaction between the SiR and FR was dominated by physical coating, with no strong chemical interactions present.

**Figure 2:**
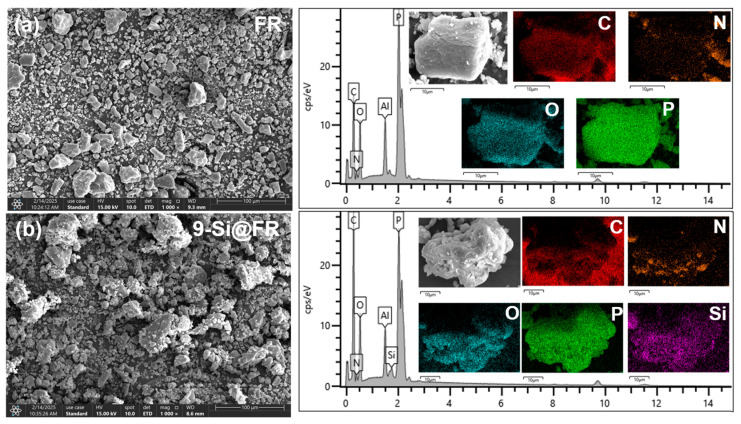
The SEM images and EDS data for the FR (**a**) and 9-SiR@FR (**b**).

To investigate the effect of the SiR shell on the microstructure of the FR, SEM and EDS were employed to systematically characterize both the uncoated FR and 9-SiR@FR. As shown in Figure 2, the FR is individually dispersed with a relatively smooth surface. After encapsulation, it was observed that 9-SiR@FR exhibited obvious particle aggregation and a rough surface, and the particle size increased significantly compared to the FR. This phenomenon indicated that SiR not only coated the surface of the FR separately but also served as an adhesive between the particles, causing them to aggregate. The surface hydrophobicity of the SiR shell enhanced the interfacial compatibility with PA66, enabling the agglomerates to form a stable contact with the matrix. This effectively hindered the migration of particles toward the surface. Moreover, the agglomerated structure further reduced the interfacial area between moisture and the FR, thereby suppressing migration. Therefore, this aggregation was believed to be beneficial for migration resistance due to the increased particle size [53,54]. The EDS results of the FR indicated that elements C, N, O, and P were distributed uniformly across the particle regions. As for the 9-SiR@FR, the maps clearly revealed that the signal of C became weak and C was replaced by Si, which was homogeneously distributed over the entire particle surface. This phenomenon provided additional evidence that the SiR shell fully encapsulated the FR particles without significantly altering the intrinsic chemical composition of the FR.

**Figure 3:**
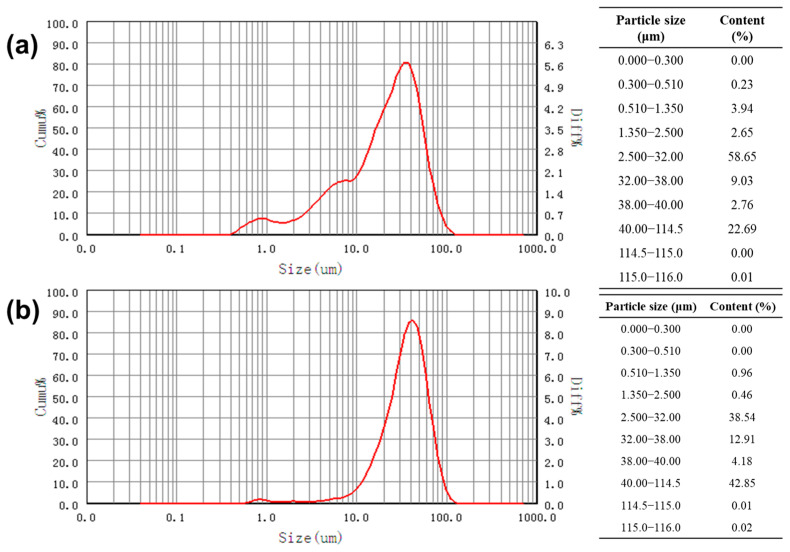
Particle size distributions of the FR (**a**) and 9-SiR@FR (**b**).

Figure 3 shows the results of the particle size distribution. It indicated a clear increase in the average particle size of the FR after SiR encapsulation. The average diameter of the FR was 26.3 μm and increased to 38.7 μm for 9-SiR@FR. Furthermore, it was found that the particle size distribution of 9-Si@FR was concentrated in the peak of 40 μm with a narrow range. Meanwhile, the content of particles smaller than 10 μm was reduced, suggesting that the small particles had agglomerated during the coating process. This observation was fully consistent with the morphology revealed by means of SEM, further confirming that SiR can effectively regulate the aggregation of FR particles.

### 3.2. Thermal Properties

**Figure 4:**
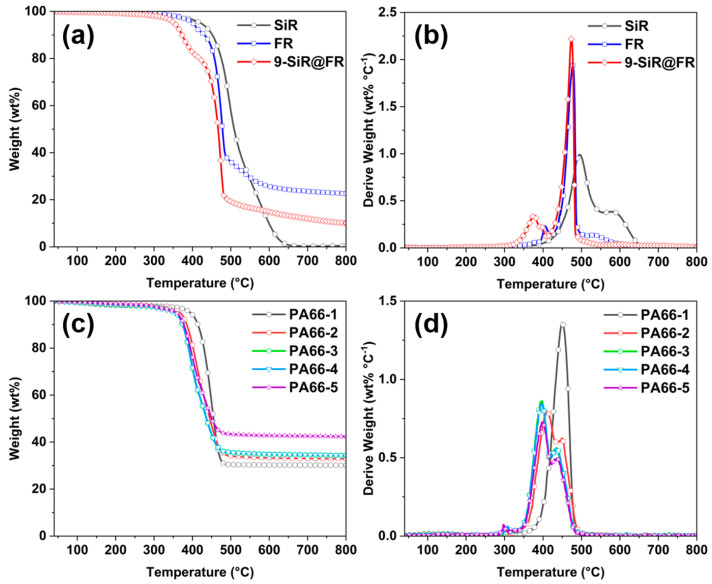
(**a**) TG and (**b**) DTG curves of the SiR, FR and 9-SiR@FR; (**c**) TG and (**d**) DTG curves of the PA66 composites under nitrogen atmosphere.

The thermal decomposition behaviors of the SiR, FR, 9-SiR@FR, and PA66 composites under nitrogen atmospheres were systematically investigated through thermogravimetric analysis (TGA). The corresponding TGA parameters were summarized in Table 3 and Figure 4. As shown in Table 3, both the SiR and FR exhibited relatively high thermal stability, with initial degradation temperatures (T_5%_) of 422 °C and 402 °C, respectively. The FR displayed two distinct thermal decomposition stages, with the max mass loss rate (R_max_) at T_max_ (R_max_) occurring at 406 °C and 477 °C, respectively. Furthermore, the FR demonstrated high char residue at 800 °C with 22.6 wt%. In contrast, although SiR also exhibited two decomposition stages at 495 °C and 579 °C, its residual char at 800 °C was very low with 0.4 wt%. The good thermal stability of SiR was primarily attributed to the restructuring of the siloxane network during decomposition. After coating with SiR, the T_5%_ of 9-SiR@FR decreased to 353 °C, suggesting that the FR promoted the early-stage degradation of SiR, which was attributed to the susceptibility of SiR to acid. However, the T_5%_ of 9-SiR@FR maintained 353 °C, above the processing temperature for PA66 of 270 °C. In terms of the flame-retardant PA66 composites, it can be observed that PA66-1 exhibited a relatively simple one-stage decomposition process, with a T_5%_ of 395 °C and char residue of 30.1 wt%. After the introduction of the FR or SiR@FR, PA66-2 to PA66-5 showed two-stage decomposition characteristics, which were closely related to the multi-step decomposition behavior of the FR. Compared with PA66-1, the T_5%_ and T_max_ of all flame-retardant samples decreased. In addition, the char residue increased steadily with increased SiR content. The residue of PA66-5 reached 42.4 wt% at 800 °C, significantly higher than that of pure PA66 and other composites. Specifically, the vinyl groups in SiR underwent radical polymerization and crosslinking initiated at elevated temperatures, forming a siloxane network. During the early stages of thermal decomposition, the polyphosphoric acid produced from the decomposition of MPP provided an acidic environment that accelerated the formation of this siloxane network. Simultaneously, acting as an acid source, the polyphosphoric acid catalyzed the hydrolysis of the PA66 molecular chains, leading to earlier decomposition. The key role of SiR lies in its physical barrier effect, which reduced the volatilization of polyphosphoric acid and provided a silicon-based structure. This promoted mutual support between the cross-linked network derived from the siloxane framework, ultimately leading to the formation of a more stable composite char layer.

### 3.3. Mechanical Properties

**Figure 5:**
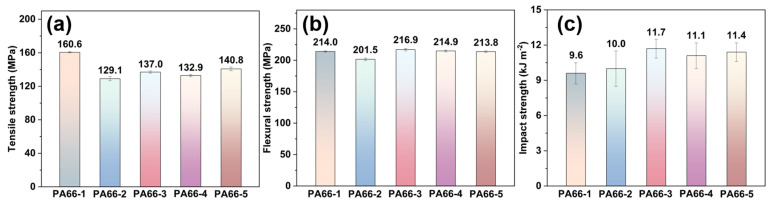
Mechanical properties of the PA66 composites: (**a**) tensile strength; (**b**) flexural strength; (**c**) impact strength.

Mechanical properties serve as critical indicators for evaluating the suitability of PA66 composites in practical application environments. A comprehensive assessment of the performance of PA66 composites was conducted through systematic testing of tensile strength, elongation at break, flexural strength, and unnotched impact strength. Detailed data are presented in Table 4 and Figure 5. The results indicated that the incorporation of the FR alone led to a significant decrease in the tensile strength of PA66, dropping from 160.6 MPa to 129.1 MPa. This reduction was primarily attributed to stress concentration induced by the FR, accompanied by the formation of increased stress defects within the PA66 matrix, which disrupted the continuity of its molecular structure. Conversely, the introduction of SiR@FR enhanced the tensile strength of the PA66 composites compared to PA66-2. Specifically, the tensile strength increased to 137.0 MPa for PA66-3 with a 6.1% improvement, 132.9 MPa for PA66-4 with a 3.1% improvement, and 140.8 MPa for PA66-5 with a 9.0% improvement. This enhancement was attributed to the SiR component, which effectively improved the interfacial compatibility, thereby mitigating the negative impact of the FR on the PA66 matrix. Meanwhile, the elongation at break for all modified composites remained at the same level, comparable to that of unmodified PA66, indicating that the overall material behavior exhibited brittle fracture characteristics. In terms of flexural strength, the addition of the FR reduced the stiffness of PA66, with PA66-2 exhibiting a value of 201.5 MPa, representing a decrease of approximately 5.8% compared to neat PA66-1. Notably, the incorporation of SiR@FR led to an improvement in flexural performance. The PA66-3 composites achieved the maximum flexural strength of 216.9 MPa. As the SiR content increased, the flexural strength of the composites showed a decreasing trend, yet remained higher than that of both neat PA66-1 and PA66-2. Furthermore, notched impact tests revealed that SiR@FR significantly enhanced impact strength. The value for PA66-3 increased to 11.7 kJ m^−2^ from 9.6 kJ m^−2^ for the unmodified PA66-1, representing an improvement of approximately 21.9%. This suggested that SiR, as a soft shell surrounding the surface of the FR, effectively absorbed energy generated during impact, thereby improving the impact strength [55]. Totally, the incorporation of SiR not only mitigated the adverse effects of the FR on the tensile and flexural strength of PA66 but also remarkably improved its impact strength. These results confirmed the critical role of SiR@FR as an efficient modifier in enhancing the overall mechanical performance of flame-retardant PA66 composites.

### 3.4. Flame Retardancy

To systematically evaluate the flame retardancy of the PA66 composites, a comprehensive analysis was conducted using the VFT and LOI. The relevant combustion test data are summarized in Table 5. PA66-1 exhibited significant flammability, with an LOI value of only 23.0%. In the VFT, it continued to burn for more than 30 s. Although no dripping was observed, it failed to achieve any UL-94 rating. After incorporating the unmodified FR, the LOI of PA66-2 increased to 28.2%, and it achieved a UL-94 V-0 rating. The average combustion time after the first ignition (t_1_) was 4.5 s, and after the second ignition (t_2_) was 2.0 s. These results indicate that the FR effectively suppressed the combustion of PA66, although the flame duration remained relatively long. Following the incorporation of SiR@FR into PA66, the LOI value of the PA66 composite was further enhanced while still maintaining the UL-94 V-0 rating. The t_1_ of PA66-3 was reduced to 1.8 s. As the content of SiR@FR increased to 6% and 9%, the LOI values rose further to 30.0% and 30.2%, respectively. In summary, SiR enhanced both the char-forming ability and flame-retardant efficiency of the FR, achieving a UL-94 V-0 rating under conditions of no dripping and high residual char.

### 3.5. Fire Behavior

**Figure 6:**
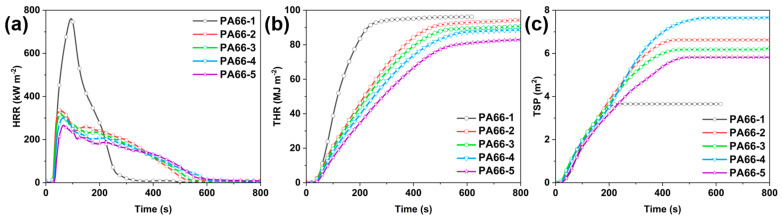
(**a**) Heat release rate (HRR), (**b**) total heat release (THR), and (**c**) total smoke production (TSP) plots of PA66 composites.

The combustion behavior of PA66 composites was systematically investigated using the cone calorimetry test, with the corresponding data summarized in Table 6 and the curves of peak heat release rate (pHRR) and total heat release (THR) presented in Figure 6. The results indicated that the incorporation of SiR slightly prolonged the ignition time (t_ig_) and the t_ig_ was extended with the increased content of SiR. Specifically, PA66-5 exhibited a t_ig_ of 30 s, which was longer than that of PA66-2 with 23 s. In terms of heat release behavior, the pHRR of PA66-1 was 769 kW m^−2^. With the addition of the FR alone, the pHRR of PA66-2 decreased to 340 kW m^−2^, representing a reduction of 56%. Further introduction of SiR@FR led to additional decreases in pHRR, with PA66-3, PA66-4, and PA66-5 exhibiting values of 322 kW m^−2^, 302 kW m^−2^, and 266 kW m^−2^, respectively. The THR of PA66-2 showed little change compared to that of PA66-1. However, with the incorporation of SiR@FR, the THR of the PA66 composites decreased noticeably. The values for PA66-3, PA66-4, and PA66-5 were 91 MJ m^−2^, 89 MJ m^−2^, and 84 MJ m^−2^, respectively, demonstrating that SiR@FR effectively suppressed heat release during combustion and exhibited excellent flame retardancy. The fire performance indexes further confirmed the enhancement in fire safety provided by SiR@FR. Moreover, the fire growth index (FIGRA), which is the maximum value of HRR(t)/t, serves as a key metric for assessing the fire-spreading behavior of materials. The FIGRA decreased consistently with the addition of SiR@FR. For instance, the FIGRA value of PA66-5 was reduced to 4.0 kW m^−2^ s^−1^, a 51% decrease compared to that of PA66-1 (8.1 kW m^−2^ s^−1^). Flame retardancy index (FRI) analysis is a key parameter for describing flame retardant efficiency. The FRI ranged between 1.4 and 4.1, indicating good flame retardancy and verifying the contribution of SiR@FR to improving the fire safety of PA66. In terms of total smoke production (TSP), the introduction of the FR alone increased the TSP from 3.7 m^2^ of PA66-1 to 6.7 m^2^ of PA66-2. With the further incorporation of SiR@FR, the TSP of PA66-5 was reduced to 5.8 m^2^, indicating that the high content of SiR@FR in this flame-retardant system effectively suppressed smoke generation. This phenomenon is primarily attributed to the trade-off between the gas-phase and condensed-phase mechanisms. On the one hand, during thermal decomposition, both the FR and SiR@FR generated phosphorus-containing free radicals and ammonia-based gases. While these contributed to flame inhibition in the gas phase via radical quenching, they also promoted incomplete combustion, leading to increased generation of soot particles. On the other hand, although SiR@FR significantly enhanced the char residue yield, the char formation process was accompanied by the pyrolysis and oxidation of volatile organic compounds, which conversely released more smoke. In conclusion, SiR@FR significantly reduced the heat release and fire spread risk of PA66 while enhancing char formation, demonstrating comprehensive fire safety improvements. However, further optimization is still required in terms of smoke suppression performance.

### 3.6. Char Analyses

**Figure 7:**
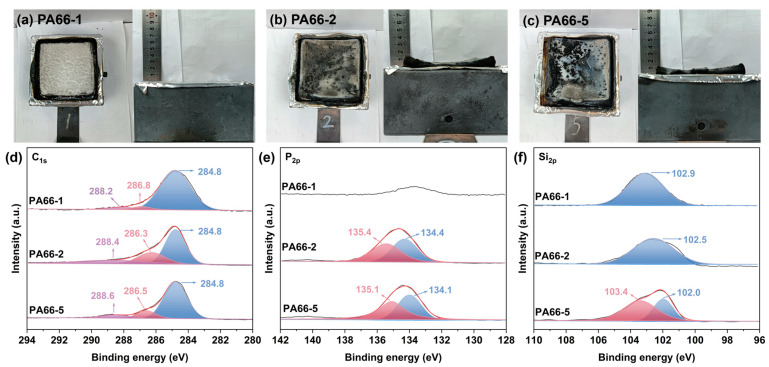
Digital photos of the char residues for (**a**) PA66-1, (**b**) PA66-2, and (**c**) PA66-5 and high-resolution XPS spectra of (**d**) C_1s_, (**e**) P_2p_, and (**f**) Si_2p_ obtained from the corresponding char residues after cone calorimeter tests.

Figure 7 presents the macroscopic morphologies of the char residues and the corresponding XPS spectra of PA66-1, PA66-2, and PA66-5 after cone calorimeter tests. The char residue of PA66-1 appeared loose and discontinuous, mainly consisting of glass fibers, which indicated severe thermal degradation of the matrix during combustion without forming effective protective char layers. There existed some differences on the surface of the char residue between PA66-2 and PA66-5. It was seen that a pale layer appeared on the surface of PA66-5, while that of PA66-2 was gray. This pale layer might be attributed to the silicon content migrating from the inner part of the char residue during combustion.

XPS analysis further elucidated the evolution of the surface chemical composition of the residues. The high-resolution XPS spectra of the char layers for PA66-1, PA66-2, and PA66-5 are displayed in Figure 7, covering the C_1s_, P_2p_, and Si_2p_ core-level regions. In the C_1s_ spectra, three samples showed a characteristic graphitic carbon peak at 284.8 eV. Peaks associated with C−O/C−N groups were observed at approximately 286.8 eV, 286.3 eV, and 286.5 eV for PA66-1, PA66-2, and PA66-5, respectively. The signals near 288.4 eV were attributed to the presence of C=O bonds. The P_2p_ spectra showed no P signal for PA66-1. For PA66-2 and PA66-5, the char layers exhibited a doublet peak centered around 134.0 eV, corresponding to P existing in phosphate and phosphite species. In the Si_2p_ spectra, peaks observed at approximately 102.9 eV, 102.5 eV, and 102.0 eV for PA66-1, PA66-2, and PA66-5, respectively, were commonly associated with SiO_2_. Notably, a new peak appeared at 103.4 eV in PA66-5, which was assigned to Si−O−C from the SiR shell, providing evidence that SiR mainly contributed to the condensed-phase mechanism [56,57,58]. This structure originated from the interaction between the high-temperature pyrolysis products of SiR and the char from the PA66 matrix. The Si−O−C network acted as a skeleton that effectively filled the pores formed by gas release during FR charring, ultimately leading to a dense silicon-reinforced char layer. This physical barrier significantly reduced the penetration of oxygen and heat into the underlying matrix and inhibited oxidative decomposition of the char. Meanwhile, the synergy between Si and P further strengthened the stability of the char layer. XPS analysis confirmed that Si existed in the form of Si−O−C/SiO_2_, which enhanced the thermal stability of the char at elevated temperatures. Phosphorus was dispersed in the char as phosphates/phosphites, forming cross-linked structures that improved the chemical stability of the char. The combined action of Si and P significantly lowered the thermal decomposition rate of the char layer. This conclusion was fully consistent with the TG results: the residual char yield of PA66-5 at 800 °C reached 42.4%, which was 9.5% higher than that of PA66-2. Moreover, the Rmax in the second degradation stage of PA66-5 was markedly lower than that of PA66-2, confirming the effective barrier and stabilizing role of the dense silicon-containing char.

### 3.7. Blooming Behavior

**Figure 8:**
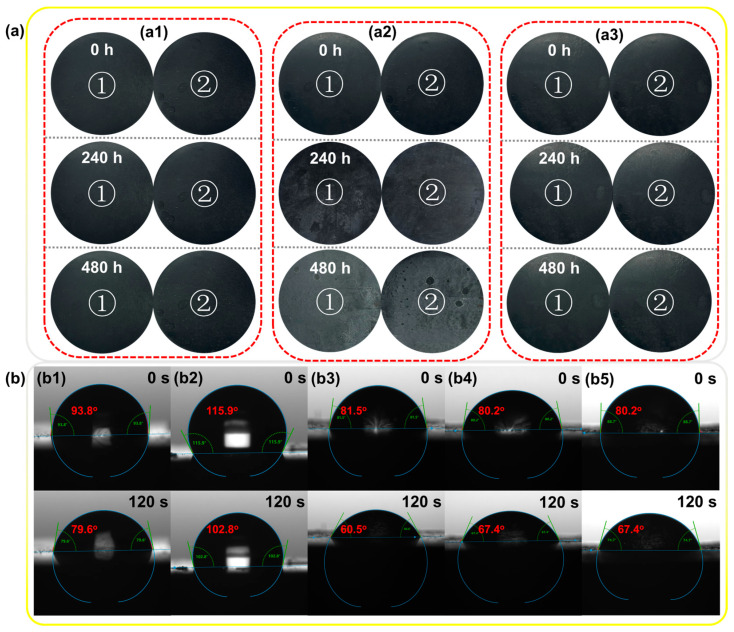
Representative digital images of the blooming behavior (**a1**–**a3**) of PA66-1, PA66-2, and PA66-5 after exposure to 85 °C and 85% relative humidity conditions, and water contact angle measurements (**b1**–**b5**) for the FR, 9-SiR@FR, PA66-1, PA66-2, and PA66-5.

To evaluate the migration resistance, all PA66 samples were subjected to an accelerated aging test at 85 °C and 85% relative humidity. As shown in Figure 8a, the surface of PA66-1 remained unchanged throughout the 480 h test. In contrast, visible blooming behavior appeared on PA66-2 after 240 h of aging, and the white frost-like deposits persisted for the entire 480 h duration. This clearly demonstrated the strong tendency of the FR to migrate to the surface under high temperature and humidity. This phenomenon was attributed to the aggressive high-temperature and high-humidity environment. The FR was water soluble, which facilitated its diffusion from the interior of the matrix to the surface under the influence of moisture. As water evaporated, these flame-retardant molecules recrystallized on the surface. This not only affected the appearance of the product but also indicated a continuous loss of flame-retardant components from the material, which led to an irreversible decline in long-term flame retardancy. In contrast, the PA66-5 samples showed no visible exudation throughout the entire 480 h aging period, and their surface remained smooth. This result confirmed that the migration tendency of the FR was significantly suppressed by the SiR coating.

**Figure 9:**
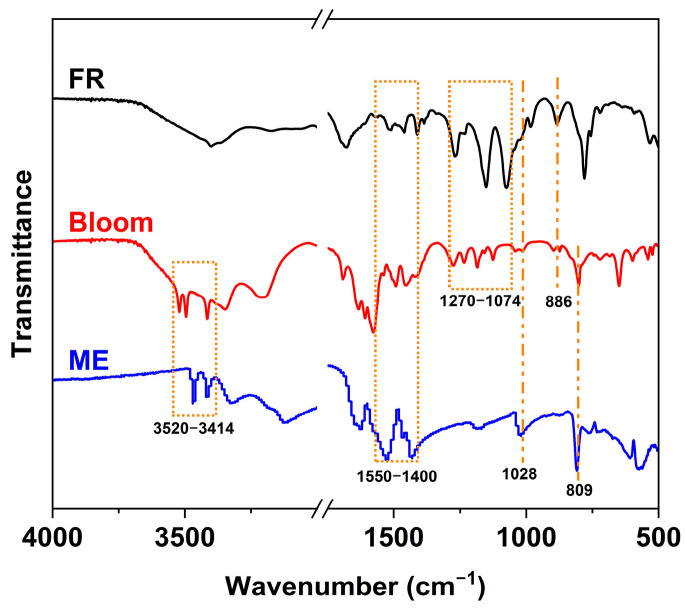
The FTIR of the bloom.

To investigate the chemical composition of the while bloom on the surface of PA66-2 samples, the bloom was analyzed via FTIR, and the results are shown in Figure 9. The results indicated that the infrared spectrum of the bloom closely matched the spectrum of melamine. Characteristic peaks were observed at approximately 3520–3414 cm^−1^, corresponding to the N−H stretching vibration. Multiple absorption peaks within the range of 1550–1400 cm^−1^ were attributed to the skeletal vibrations of C=N and C−N. The characteristic peaks at around 1028 cm^−1^ and 809 cm^−1^ were assigned to the triazine ring vibration. Meanwhile, characteristic peaks were also observed in the bloom spectrum at 1270 cm^−1^ and 1074 cm^−1^, which were assigned to the P=O and P−O stretching vibrations, respectively. The absorption near 886 cm^−1^ corresponded to the P−O−P vibration. These features were consistent with those observed in the FR spectrum. The above results demonstrated that the surface bloom primarily consisted of melamine (ME) derived from MPP and phosphorous salts derived from the decomposition products or oligomer in the FR.

Water contact angle measurements provided critical insights into the blooming behavior of the PA66 composites. As shown in Figure 8b, the FR exhibited strong hydrophilicity, with an initial contact angle of 93.8° that decreased to 79.6° after 120 s. In contrast, 9-SiR@FR exhibited enhanced hydrophobicity with an initial contact angle of 115.9° and remained at 102.8° after 120 s, confirming the effectiveness of the SiR shell in improving water resistance. As for the PA66 composites, PA66-1 displayed a contact angle of 81.5°, reflecting the inherent wettability of the PA66 matrix. The PA66 that incorporates the FR led to a slightly lower contact angle of 80.2°, suggesting increased surface hydrophilicity after the introduction of the FR. Notably, PA66-5, containing 9-SiR@FR, exhibited a contact angle of 88.7°, even more than that of PA66-1. To sum up, the anti-blooming mechanism can be summarized in two aspects. On the one hand, the SiR shell enhanced the hydrophobicity of the FR, thereby isolating it from direct moisture contact and preventing its water-induced migration to the polymer surface. On the other hand, the SiR coating enhanced the surface hydrophobicity of the composites, thereby effectively suppressing water penetration. As a result, the blooming behavior was suppressed.

## 4. Conclusions

In this work, a core–shell structured SiR@FR was successfully fabricated via a solution deposition method, which effectively addressed the key technical challenge of poor migration resistance and surface whitening of conventional ADP and MPP flame retardants in PA66 composites. The results demonstrated that the introduction of SiR significantly increased the migration resistance of the FR by improving the hydrophobicity of the FR and PA66 composites. After aging at 85 °C and 85% relative humidity for 480 h, no surface whitening was observed for PA66-5. In terms of flame retardancy, PA66-5 exhibited an LOI value of 30.2%, achieved a UL-94 V-0 rating, and pHRR was reduced to 266 kW m^−2^. Our analysis suggested that the superior fire performance of PA66-5 originated from the formation of a coherent, silicon-reinforced char barrier in the condensed phase during combustion. Regarding mechanical properties, the incorporation of SiR@FR improved the impact strength of the PA66 composite by approximately 21% due to its elasticity and improved compatibility. This work has established a robust and practical strategy for engineering high-performance PA66 composites that combine long-term migration resistance with good fire safety and mechanical properties, holding significant promise for advanced applications in demanding environments.

## Figures and Tables

**Table 1:** Formulas of the FR and SiR@FR.

Sample	ADP (g)	MPP (g)	MVPS (g)	DHBP-95 (g)	Ethyl Acetate (mL)
FR	150	50	-	-	-
3-SiR@FR	150	50	6	0.12	100
6-SiR@FR	150	50	12	0.24	100
9-SiR@FR	150	50	18	0.36	100

**Table 2:** Formulas of the flame-retardant PA66 composites.

Sample	PA66-1	PA66-2	PA66-3	PA66-4	PA66-5
PA66	67.6	51.6	51.6	51.6	51.6
FR	-	16	-	-	-
3-SiR@FR	-	-	16	-	-
6-SiR@FR	-	-	-	16	-
9-SiR@FR	-	-	-	-	16
Glass fibers	30	30	30	30	30
Black master batch	2	2	2	2	2
Antioxidant168	0.2	0.2	0.2	0.2	0.2
Antioxidant1098	0.2	0.2	0.2	0.2	0.2

**Table 3:** Typical parameters obtained from TG analyses under nitrogen atmosphere.

Samples	T_5%_ (°C)	T_max_ (°C)	R_max_ (wt% °C^−1^) ^a^	Residue at 800 °C (wt%)
SiR	422	T_max1_ = 495/T_max2_ = 579	R_max1_ = 0.99/R_max2_ = 0.39	0.4
FR	402	T_max1_ = 406/T_max2_ = 477	R_max1_ = 0.23/R_max2_ = 1.94	22.6
9-SiR@FR	353	T_max1_ = 378/T_max2_ = 474	R_max1_ = 0.34/R_max2_ = 2.22	10.1
PA66-1	395	T_max1_ = 450	R_max1_ = 1.36	30.1
PA66-2	368	T_max1_ = 411/T_max2_ = 450	R_max1_ = 0.79/R_max2_ = 0.62	32.9
PA66-3	357	T_max1_ = 398/T_max2_ = 436	R_max1_ = 0.86/R_max2_ = 0.56	34.3
PA66-4	354	T_max1_ = 397/T_max2_ = 436	R_max1_ = 0.85/R_max2_ = 0.56	34.4
PA66-5	358	T_max1_ = 398/T_max2_ = 435	R_max1_ = 0.73/R_max2_ = 0.50	42.4

^a^ R_max_: Mass loss rate at T_max_.

**Table 4:** Mechanical properties of the PA66 composites.

Samples	Tensile Strength (MPa)	Elongation at Break (%)	Flexural Strength (MPa)	Notched Impact Strength (kJ m^−2^)
PA66-1	160.6 ± 0.8	3.2 ± 0.2	214.0 ± 1.0	9.6 ± 0.9
PA66-2	129.1 ± 2.6	3.1 ± 0.3	201.5 ± 2.1	10.0 ± 1.5
PA66-3	137.0 ± 1.3	3.0 ± 0.1	216.9 ± 1.9	11.7 ± 0.8
PA66-4	132.9 ± 1.4	3.1 ± 0.2	214.9 ± 1.5	11.1 ± 1.1
PA66-5	140.8 ± 2.2	2.9 ± 0.1	213.8 ± 1.4	11.4 ± 0.8

**Table 5:** Key data of LOI and UL-94 tests.

Samples	LOI (%)	UL-94 (1.6 mm)			
		t_1_ (s) ^a^	t_2_ (s) ^a^	Dripping	Rating
PA66-1	23.0 ± 0.5	>30.0	-	No	NR
PA66-2	28.2 ± 0.3	4.5 ± 2.5	2.0 ± 0.8	No	V-0
PA66-3	29.6 ± 0.5	1.8 ± 0.5	0.8 ± 1.0	No	V-0
PA66-4	30.0 ± 0.2	1.5 ± 0.6	2.0 ± 1.1	No	V-0
PA66-5	30.2 ± 0.4	1.5 ± 1.0	1.8 ± 1.5	No	V-0

^a^ Average burning duration after the first (t_1_) and second ignition (t_2_).

**Table 6:** Cone calorimeter data of the PA66 composites.

Samples	t_ig_ (s)	pHRR (kW m^−2^)	THR (MJ m^−2^)	t_pHRR_ (s)	FIGRA (kW m^−2^ s^−1^)	FRI ^a^	TSP (m^2^)	Residue (wt%)
PA66-1	24 ± 1	769 ± 12	96 ± 3	95 ± 3	8.1	-	3.7 ± 0.5	28.3 ± 0.2
PA66-2	23 ± 3	340 ± 15	95 ± 4	53 ± 2	6.4	1.3	6.7 ± 0.8	33.3 ± 0.3
PA66-3	23 ± 1	322 ± 10	91 ± 2	55 ± 3	5.9	1.4	6.4 ± 0.6	36.1 ± 0.4
PA66-4	27 ± 2	302 ± 11	89 ± 1	68 ± 1	4.4	2.0	7.7 ± 0.4	36.6 ± 0.6
PA66-5	30 ± 1	266 ± 12	84 ± 1	65 ± 3	4.0	4.1	5.8 ± 0.6	36.4 ± 0.3

^a^ FRI = (THR × pHRR/t_ig_) neat PA66 /(THR × pHRR/t_ig_) flame-retardant PA66 composites.

## Data Availability

Data are contained within the article.
